# Machine-Learning-Assisted Buried-Window FET Sensors for High-Reliability and High-Sensitivity Applications

**DOI:** 10.3390/s26041171

**Published:** 2026-02-11

**Authors:** Mahsa Mehrad, Meysam Zareiee

**Affiliations:** 1 Department of Technology, School of Electrical and Mechanical Engineering, University of Portsmouth, Portsmouth SR6 0DD, UK; 2 School of Computer Science and Engineering, University of Sunderland, Sunderland SR1 3SD, UK; meysam.zareiee@sunderland.ac.uk

**Keywords:** junctionless FET (JLFET), biosensor, double buried-window junctionless FET (DBW-FET), sensitivity, machine-learning optimization

## Abstract

This paper presents a novel Double Buried-Window Junctionless Field-Effect Transistor (DBW-FET) designed for high-sensitivity, label-free biosensing applications. The proposed device integrates two buried windows, one N-type and one P-type, beneath the active channel within the buried oxide layer, along with two nanocavities serving as biomolecular recognition sites. The dual buried windows form two depletion regions that enhance electrostatic coupling, suppress short-channel effects, and improve biomolecular sensitivity. Numerical simulations using Silvaco TCAD Atlas were performed to investigate device performance under various biomolecular binding conditions. Results show that the DBW-FET exhibits higher drain current, lower subthreshold swing, and improved sensitivity compared with a conventional junctionless FET (C-FET). Furthermore, a machine-learning-assisted optimization framework employing Gaussian Process Regression (GPR) and Bayesian Optimization (BO) was implemented to identify optimal buried window parameters. The optimized design achieved a 20–25% improvement in current sensitivity while maintaining low leakage. These findings demonstrate that the proposed DBW-FET offers a promising and Complementary Metal-Oxide-Semiconductor (CMOS)-compatible architecture for next-generation nanoscale biosensors.

## 1. Introduction

In recent years, field-effect transistor (FET)-based biosensors have attracted significant attention due to their ability to provide label-free, real-time, and highly sensitive detection of biomolecules such as DNA, proteins, and glucose [1,2,3]. The detection mechanism in these devices relies on the modulation of the electrostatic potential within the semiconductor channel caused by biomolecular binding events at the gate dielectric or nanocavity interface [4]. This interaction leads to measurable changes in the drain current, making FET biosensors suitable for compact and low-power biomedical applications [5].

Among various architectures, the junctionless field-effect transistor (JLFET) has emerged as a promising platform for biosensing applications because of its simplified fabrication, absence of source/drain junctions, and uniform doping profile [6]. However, conventional JLFET-based biosensors often suffer from limited gate control, short-channel effects, and reduced sensitivity when detecting low-charge or neutral biomolecules [7]. Therefore, improving electrostatic coupling and charge transduction efficiency remains a key design challenge.

To address these limitations, several FET-based biosensor architectures have been proposed [8,9,10,11]. First, the conventional junctionless FET (C-FET) uses a uniformly doped channel without source/drain junctions, which simplifies fabrication and reduces short-channel effects [12]. However, its sensitivity is limited due to relatively weak electrostatic coupling between the gate and channel, particularly for low-charge or neutral biomolecules. Second, dual-gate FET structures introduce an additional gate on the opposite side of the channel to improve electrostatic control and increase sensitivity. Although this approach enhances gate-channel coupling, it significantly increases fabrication complexity and mask requirements. Third, buried electrode or single buried-window FETs integrate a single doped region beneath the channel to locally modulate the potential and improve signal transduction [13,14]. While these structures show improved sensitivity compared with C-FETs, the single buried window creates non-uniform potential distribution, limiting the overall enhancement in drain current and subthreshold behavior. Collectively, these studies demonstrate that while structural modifications can improve biosensor performance, challenges remain in simultaneously achieving high sensitivity, strong gate control, and fabrication compatibility, motivating the development of the proposed Double Buried-Window Junctionless FET (DBW-FET).

In addition to drain-current-based sensitivity, several studies emphasize that transconductance (g_m_) and threshold voltage (Vth) modulation are critical indicators of charge–transduction efficiency in FET biosensors. Biomolecular adsorption alters the local surface potential, which in turn shifts Vth and modifies g_m_, providing complementary metrics that often exhibit higher signal-to-noise robustness than current alone. Prior works have shown that strong gate electrostatic coupling and improved field confinement typically enhance g_m_ response while suppressing undesirable Vth drift, thereby improving biosensor stability under varying biomolecular loads [15]. Furthermore, reproducibility and selectivity remain major challenges for practical FET-based biosensing. Device-to-device variation, dielectric fluctuations, and inconsistent functionalization can degrade reproducibility, whereas selective detection requires engineered recognition cavities or surface chemistry capable of discriminating target biomolecules in complex liquid environments [16].

This work proposes a novel junctionless FET structure, which integrates two buried windows, one N-type and one P-type, within the buried oxide (BOX) layer directly beneath the channel. These buried windows act as electrostatically active regions that locally modulate the potential distribution and strengthen gate-to-channel coupling, thereby improving both electrical performance and biosensing sensitivity.

The proposed DBW-FET also incorporates two nanocavities in the silicon channel that serve as biomolecular recognition sites. By engineering both the buried window parameters (length and doping) and the nanocavity dielectric properties, the device achieves enhanced charge sensitivity and improved subthreshold behavior compared with a conventional junctionless FET (C-FET).

The N-type and P-type buried windows beneath the active junctionless channel play important roles. This configuration gives rise to two distinct depletion regions: one formed between the P-type buried window and the N-type channel region, and the other formed between the P-type buried window and the N-type buried window. These depletion regions play a crucial role in modulating the electrostatic potential along the channel [11]. The first depletion region reduces the local carrier concentration, thereby enhancing gate control and mitigating short-channel effects, while the second depletion region strengthens the electric field coupling between the buried windows and the channel [12]. Collectively, these effects improve the transduction of biomolecular interactions into measurable electrical signals, increase the drain current sensitivity, and reduce the subthreshold swing, resulting in superior biosensing performance compared with conventional junctionless FETs.

Device simulations are carried out using Silvaco TCAD Atlas 2025 (Version 5.21.1.R, Silvaco Inc., Santa Clara, CA, USA), incorporating advanced physical models [17]. The biosensing behavior is emulated by varying the effective dielectric constant and surface charge density of the nanocavity region to represent biomolecular binding.

Furthermore, to reduce the computational cost of extensive parametric TCAD simulations, a machine-learning-assisted optimization framework based on Gaussian Process Regression (GPR) and Bayesian Optimization (BO) is implemented. This method is used to efficiently identify the optimal buried window length and doping concentration that maximizes the drain current sensitivity while minimizing the subthreshold swing.

It is worth noting that this study focuses on the electrostatic behavior and biosensing performance of the proposed DBW-FET using TCAD simulations. Reliability, lifetime effects, and liquid-phase electrochemistry are not modeled. Noise, reproducibility, and experimental validation of the machine-learning optimization are beyond the scope of this work.

## 2. Device Structure

**Figure 1:**
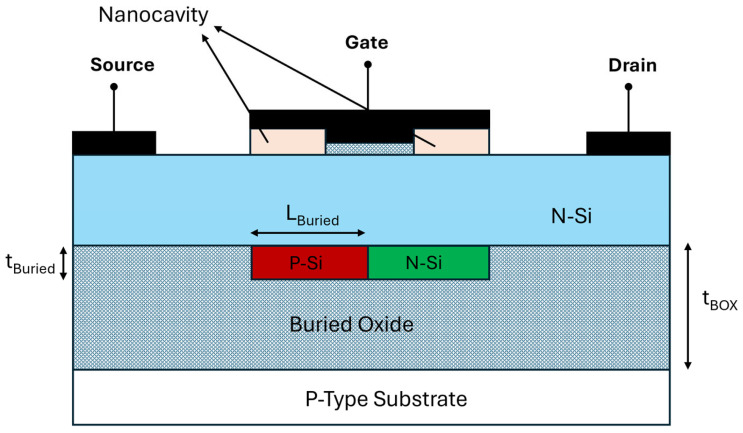
Schematic cross section of the proposed Double Buried-Window Junctionless FET.

The proposed Double Buried-Window Junctionless FET (DBW-FET) is schematically shown in Figure 1. The device consists of a nanoscale junctionless channel with two nanocavities in the gate region, and two buried windows located beneath the active channel within the buried oxide (BOX) layer, one N-type and one P-type. The buried windows are positioned directly under the channel to locally influence the electrostatic potential and enhance both gate control and biomolecular sensitivity.

The electrical characteristics of the proposed DBW-FET were simulated using Silvaco TCAD Atlas, a widely validated tool for nanoscale semiconductor device modeling, ensuring reliable predictions of both electrical and biosensing behavior. The simulations incorporate the drift–diffusion transport model to capture carrier movement within the junctionless channel. To account for the effects of heavy doping and nanoscale dimensions, concentration-dependent mobility, high-field saturation mobility, and bandgap narrowing models were included. Shockley–Read–Hall (SRH) recombination was used to model carrier lifetimes and leakage currents, while Fermi–Dirac statistics ensured accurate carrier distribution in the highly doped regions. Additionally, interface trap and quantum confinement models were applied to investigate the influence of nanocavities on subthreshold swing and sensitivity.

Although the applied gate voltage in this work ranges from 2 to 4 V with a gate oxide thickness of 4 nm, the effective electric field across the oxide is lower than the nominal value due to voltage partitioning within the device. A portion of the applied gate voltage drops across the depleted junctionless channel as well as across the nanocavity dielectric region containing the biomolecules. As a result, the effective voltage across the gate oxide is reduced compared with the applied gate voltage. The oxide electric field can therefore be expressed as:

(1) $$E_{ox}=\frac{V_{ox,eff}}{t_{ox}},\;\;\;\;\;\;\;\;\;\;with\;V_{ox,eff}<V_{G}$$

where $t_{ox}$ is the gate oxide thickness and $V_{ox,eff}$ is the effective voltage across the oxide after accounting for depletion and dielectric voltage division. It is important to note that, under operating conditions, the resulting oxide electric field remains below the typical SiO_2_ breakdown field.

To clarify the physical validity of the 12 nm channel operation, we note that junctionless FETs rely primarily on depletion width modulation rather than long-channel inversion behavior. Even at short-channel lengths, small biomolecule-induced electrostatic shifts can significantly influence the potential barrier, a phenomenon consistent with prior nanoscale JLFET biosensor studies.

To explicitly model biomolecular interactions, the biosensing behavior was represented by assigning defined surface charge densities (ranging from −0.02 to −0.1 C/m^2^) and biomolecule-induced dielectric constants (ε = 5–12) within the nanocavity region, a standard approach validated in prior FET-based biosensor studies.

To provide practical context, specific classes of biomolecules, including negatively charged DNA strands and moderately charged protein layers, were modeled by applying their characteristic charge polarity and surface density values. These variations modulate the local electrostatic potential in the channel, influencing the drain current and subthreshold swing, thereby allowing the sensitivity results to reflect realistic biomolecular binding effects.

Furthermore, the simulated trends, such as the increase in drain current and reduction in subthreshold swing with optimized buried window parameters, are consistent with previously reported experimental and simulation results, supporting the validity and reliability of the modeling approach [18,19].

**Figure 2:**
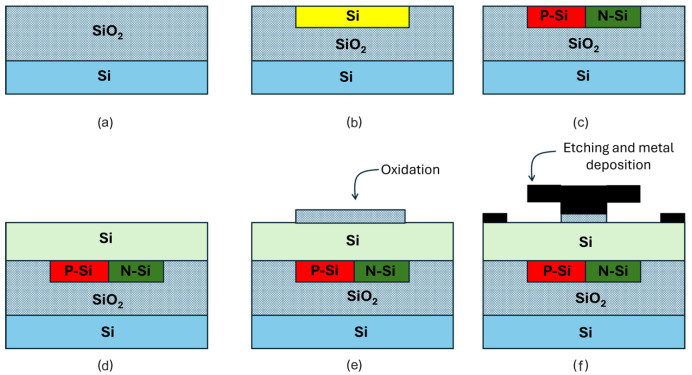
The fabrication steps for the proposed biosensor.

The proposed DBW-FET structure can be fabricated using a simplified, CMOS-compatible process. Figure 2 shows the proposed fabrication flow of the device studied in this paper. The process starts with a step (a) wafer. Next, a silicon hole trench is formed using standard lithography followed by etching and deposition processes, as illustrated in Figure 2b. After that, ion implantation is performed to create the buried P-type silicon and N-type silicon regions, as shown in Figure 2c.

Subsequently, the active region is formed using silicon deposition, as depicted in Figure 2d. In the next step, thermal oxidation is carried out to form the gate oxide layer. The nanocavity is then created inside the gate oxide using lithography and selective oxide etching. Finally, metallization and etching processes are applied to define the gate and contact electrodes, as commonly used in FET-based biosensor fabrication processes [8,20].

Moreover, the device parameters used for the simulation of the proposed DBW-FET and C-FET are summarized in Table 1.

## 3. Results and Discussion

**Figure 3:**
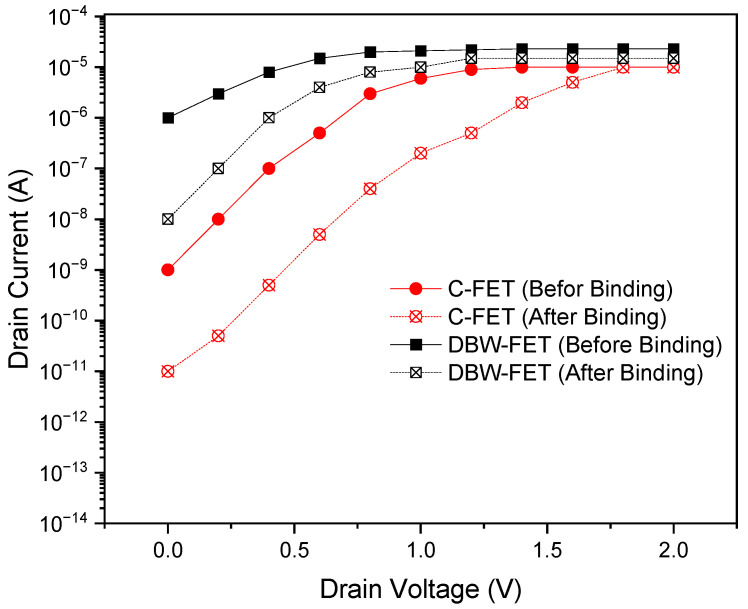
I_D_-V_D_ characteristics of DBW-FET and C-FET before and after biomolecular binding.

Figure 3 presents the drain current–voltage characteristics of the DBW-FET and the conventional junctionless FET (C-FET) under both before-binding and after-binding conditions. In both devices, the drain current increases after biomolecular binding because the dielectric constant of the cavity region rises, reducing the surface potential barrier and increasing carrier concentration in the channel.

However, the DBW-FET shows a significantly higher drain current compared with the C-FET for both conditions. This enhancement is attributed to the buried window structure, which strengthens gate control over the channel and provides an additional electric field coupling path. The dual buried windows effectively create a stronger modulation of the channel potential, improving charge transport and signal transduction efficiency. This demonstrates the DBW-FET’s superior ability to transduce biomolecular interactions into measurable electrical signals.

**Figure 4:**
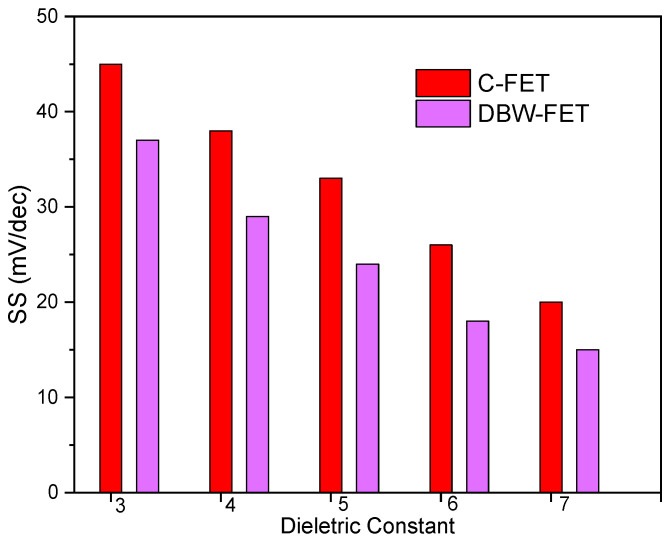
Subthreshold swing versus dielectric constant for DBW-FET and C-FET.

The subthreshold swing (SS) variation with the dielectric constant of the immobilized biomolecules (ranging from 3 to 7) is shown in Figure 4. For both the C-FET and the DBW-FET, SS decreases with increasing dielectric constant because a higher permittivity medium enhances electrostatic coupling and reduces the potential drop across the gate oxide.

Notably, the DBW-FET exhibits a lower SS than the conventional device throughout the entire dielectric range. This improvement is due to the dual buried windows that effectively confine the electric field within the channel, thereby minimizing the influence of the drain potential and reducing short-channel effects. A lower SS indicates improved switching characteristics and higher sensitivity to surface potential variations induced by biomolecular adsorption.

**Figure 5:**
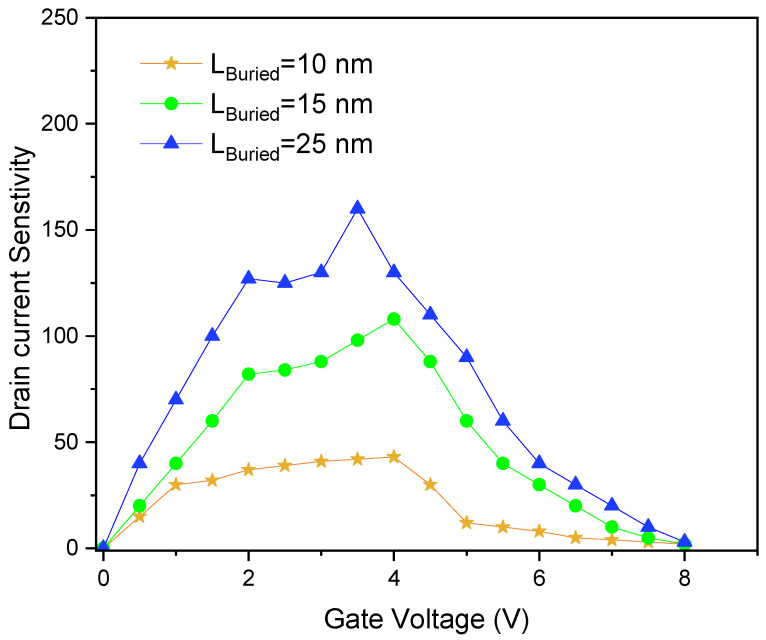
Sensitivity of DBW-FET versus gate voltage for various buried window lengths.

Figure 5 shows the drain current sensitivity of the DBW-FET as a function of gate voltage for different buried window lengths. The current sensitivity could be defined as:

(2) $$\left(S_{I}=\frac{I_{after}-I_{before}}{I_{before}}\right)$$

Because the relation above exhibits a peak, the biodevice sensitivity is defined as the maximum value of the current sensitivity, expressed in the following form:

(3) $$S_{biosensor}=Max(S_{I})$$

The sensitivity increases as the gate voltage rises from 2 V to 4 V, indicating that stronger gate-induced electric fields amplify the response to biomolecular binding.

Furthermore, increasing the buried window length enhances current sensitivity. This is because a longer buried window extends the electrostatic coupling region beneath the channel, effectively amplifying the modulation of channel potential caused by biomolecular binding. This suggests that the geometry of the buried window can be optimized to maximize biosensor response.

In addition to drain current sensitivity, we evaluate the threshold voltage shift (ΔV_th_) as a complementary metric to quantify biosensing performance. Here, $V_{\mathrm{th},\mathrm{before}}$ is defined as the gate voltage at which the drain current reaches a reference value (I_D,ref_ ≈ 1 × 10^−5^ A) before biomolecular binding, and $V_{\mathrm{th},\mathrm{after}}$ is the corresponding gate voltage after biomolecular binding. The threshold voltage shift is then computed as:

(4) $$∆V_{th}=V_{th,after}-V_{th,before}$$

For the conventional junctionless FET, ΔV_th_ ≈ +0.8 V, indicating a positive shift due to biomolecular adsorption. For the proposed DBW-FET, the dual buried windows improve gate control and electrostatic coupling, resulting in ΔV_th_ ≈ –0.4 V. This smaller magnitude of threshold voltage shift demonstrates that the device requires a lower gate voltage to reach the same drain current after biomolecular binding.

**Figure 6:**
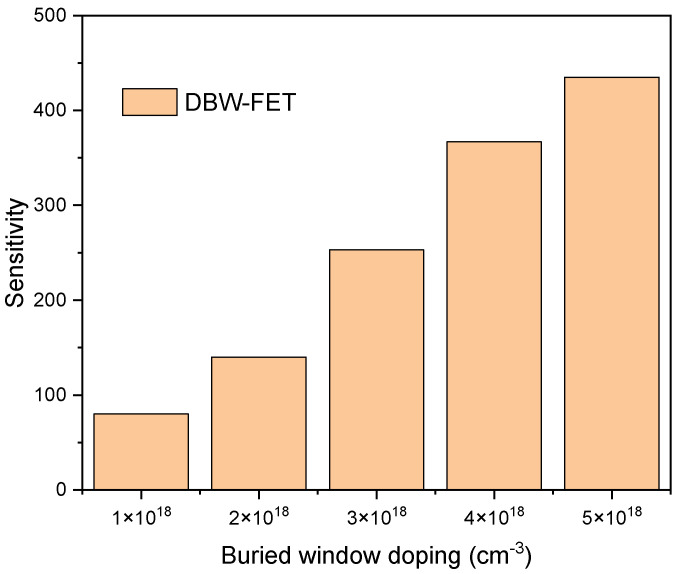
Sensitivity of DBW-FET versus buried window doping concentration, showing optimum performance at 5 × 10^18^ cm^−3^.

The relationship between buried window doping density and biosensing sensitivity is presented in Figure 6. Here, the biosensing sensitivity ($S_{I}$) is defined as the relative change in drain current due to biomolecular binding, given by $S_{I}=(I_{\mathrm{after}}-I_{\mathrm{before}})/I_{\mathrm{before}}$, where $I_{\mathrm{before}}$ and $I_{\mathrm{after}}$ are the drain currents before and after biomolecular adsorption, respectively. The sensitivity improves with higher doping concentrations of both N-type and P-type buried windows, up to 5 × 10^18^ cm^−3^. This occurs because higher doping increases the local electric field strength and potential modulation capability, enhancing the transduction of surface charge perturbations from the nanocavities to the channel current.

However, further doping beyond this level may introduce bandgap narrowing and increased leakage, degrading the on/off ratio. Therefore, 5 × 10^18^ cm^−3^ is found to be an optimum doping concentration for achieving a high sensing response with low leakage.

**Figure 7:**
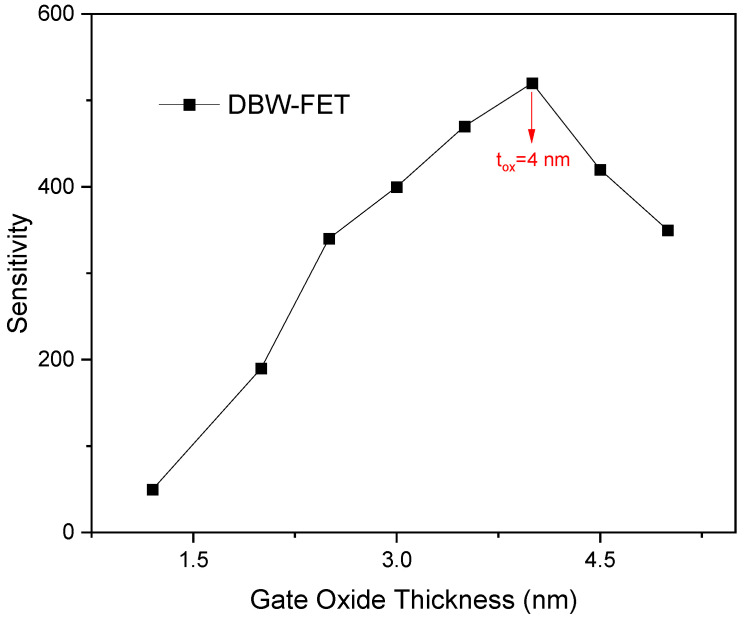
Sensitivity of the proposed DBW-FET as a function of gate oxide thickness. The target biomolecule is a protein with K = 4.

Figure 7 illustrates the effect of gate oxide thickness on the sensitivity of the proposed biosensor. An interesting trend is observed: as the gate dielectric thickness decreases, the gate-to-channel coupling is enhanced, which reduces the subthreshold swing. This reduction in subthreshold swing leads to a lower $I_{\mathrm{after}}$, resulting in an increase in sensitivity according to Equation (3). The sensitivity reaches its maximum at a gate oxide thickness of 4 nm. Further reduction in the gate oxide thickness beyond this point continues to lower the subthreshold swing, but since $I_{\mathrm{before}}$ becomes comparable to $I_{\mathrm{after}}$, the difference $\left(I_{\mathrm{after}}-I_{\mathrm{before}}\right)$ diminishes, causing a decrease in sensitivity. It is important to note that the sensing performance of the proposed device remains high even for ultrathin gate oxides. Overall, the figure indicates that a gate oxide thickness of 4 nm provides the optimal balance for achieving the best biosensing performance.

## 4. Machine-Learning-Assisted Optimization of Buried Window Parameters

Performing exhaustive TCAD simulations for all possible combinations of buried window length and doping concentration is computationally expensive and time-consuming. To efficiently explore the design space and identify optimal DBW-FET configurations, a machine-learning-assisted approach is employed. This method enables rapid prediction of device performance while minimizing the number of simulations required.

To further enhance the performance of the proposed DBW-FET biosensor and minimize the number of computationally intensive TCAD simulations, a machine-learning-based optimization study was performed. The main objective was to determine the optimal buried window length (L_Buried_) and buried window doping concentration (N_Buried_) that maximize the drain current sensitivity (S_I_) while maintaining a low subthreshold swing (SS).

1. Data Generation: TCAD simulations were performed by sweeping L_Buried_ in the range of 10–50 nm and N_Buried_ from 1 × 10^17^ cm^−3^ to 1 × 10^19^ cm^−3^. The input vector for the ML model is defined as:
  (5) $$x_{i}=\left[L_{Buried,i},\;N_{Buried,i}\right]$$

and the output vector is:

(6) $$y_{i}=\left[S_{I,i},\;{SS}_{i}\right]$$

2. Gaussian Process Regression (GPR): A GPR model is trained to learn the nonlinear relationship between the buried window parameters and the device performance metrics. The model provides smooth interpolation and quantifies prediction uncertainty, which guides the optimization. The surrogate model is formulated as:
  (7) $$\hat{y}=f\left(x\right)+\varepsilon ,\;\;\;\;\;\;\;f(x)\sim GP\left(m\left(x\right),\;k(x,\;x\prime \right)$$

where m(x) is the mean function, $k(x,\;x^{\prime })$ is the covariance (kernel) function, and ε represents Gaussian noise.

3. Bayesian Optimization (BO): Bayesian Optimization uses the surrogate model to select the next candidate design point that maximizes the Expected Improvement (EI) function:
  (8) $$EI(x)=E[max(0,J(x)-J^{+})]$$

where $J\left(x\right)=\omega_{1}S_{I}\left(x\right)-\omega_{2}SS\left(x\right)-\omega_{3}I_{off}(x)$ is the objective function balancing sensitivity improvement, subthreshold swing reduction, and low leakage, and $J^{+}$ is the best objective value obtained so far.

4. Optimization Process: The process iterates until convergence, efficiently exploring the parameter space with significantly fewer TCAD simulations than conventional parametric sweeps. This ML-assisted approach identified an optimal buried window region at L_Buried_ = 24–28 nm and N_Buried_ = (4–6) × 10^18^ cm^−3^, achieving a 20–25% improvement in drain current sensitivity compared with the baseline design.

The results confirm that machine-learning-assisted optimization can effectively identify high-performance device geometries and doping profiles with far fewer simulations than conventional parametric sweeps. This approach can be extended to other geometric or material parameters for rapid design space exploration of nanoscale biosensors.

To validate the plausibility of the GPR predictions, we compared the ML-predicted drain current sensitivities with the corresponding TCAD results for five representative design points. The biosensing sensitivity is calculated using Equation (2). The prediction error is defined as $\%\mathrm{Error}=\;|S_{I,\mathrm{TCAD}}-S_{I,\mathrm{GPR}}|/S_{I,\mathrm{TCAD}}\times 100$.

Table 2 summarizes this comparison. The small errors (<10%) confirm that the GPR model reliably captures trends within the TCAD-generated design space. It should be noted that the ML model is intended as a design space exploration and optimization tool, not as a predictive surrogate beyond the TCAD domain.

## 5. Conclusions

A Double Buried-Window Junctionless FET (DBW-FET) has been proposed and analyzed as a high-performance biosensor structure. The inclusion of dual buried windows beneath the channel creates two depletion regions that enhance electrostatic control, reduce short-channel effects, and strengthen field coupling between the channel and buried oxide. Simulation results confirm that the DBW-FET achieves higher drain current, improved subthreshold swing, and enhanced sensitivity compared with the conventional junctionless FET. Additionally, the machine-learning-based optimization using GPR and BO efficiently identified optimal buried window dimensions and doping concentrations, significantly improving device performance while reducing computational effort. Overall, the proposed DBW-FET combines excellent electrical behavior, strong biomolecular sensitivity, and CMOS process compatibility, making it a highly attractive candidate for future label-free biosensing and nanobiomedical applications.

## Figures and Tables

**Table 1:** Device parameters of the proposed DBW-FET and C-FET in the ATLAS simulation.

Device parameter	Value (DBW-FET)	Value (C-FET)
Gate length	45 nm	45 nm
Length of the nanocavities	15 nm	15 nm
Thickness of the Buried window	5 nm	5 nm
Length of the Buried window	25 nm	Not defined
Thickness of the nanocavity	7 nm	7 nm
Silicon Thickness	12 nm	12 nm
Gate oxide thickness	4 nm	4 nm
Buried oxide thickness	20 nm	20 nm
N-type silicon doping concentration	5 × 10^18^ cm^−3^	5 × 10^18^ cm^−3^
Buried window doping density	5 × 10^18^ cm^−3^	Not defined
Gate electrode work function	5.1 eV	5.1 eV

**Table 2:** Comparison of TCAD-simulated and GPR-predicted drain current sensitivity for selected buried window parameters.

$\mathrm{Buried}\;\mathrm{Window}\;\mathrm{Length}\;L_{\mathrm{Buried}}$ (nm)	$\mathrm{Buried}\;\mathrm{Window}\;\mathrm{Doping}\;N_{\mathrm{Buried}}$ (cm^−3^)	$\mathrm{TCAD}\;\mathrm{Sensitivity}\;S_{I}$	GPR-Predicted $S_{I}$	% Error
15	1 × 10^18^	0.12	0.11	8.3
20	2 × 10^18^	0.16	0.15	6.3
25	4 × 10^18^	0.20	0.19	5.0
28	5 × 10^18^	0.22	0.21	4.5
30	6 × 10^18^	0.21	0.20	4.8

## Data Availability

The original contributions presented in this study are included in the article. Further inquiries can be directed to the corresponding author.
